# Extensive slow-flow vascular malformation of the left lower limb

**DOI:** 10.11604/pamj.2026.53.78.49805

**Published:** 2026-02-12

**Authors:** Riya Yadav, Pratap Singh Parihar

**Affiliations:** 1 Department of Radiology, Datta Meghe Institute of Higher Education and Research, Wardha, India

**Keywords:** Slow-flow lesion, phleboliths, lower limb

## Image in medicine

A 5-year-old boy presented with progressive swelling and hypertrophy of the left lower limb since early childhood. The swelling was painless and gradually increased in size, with no history of trauma or systemic symptoms. Magnetic resonance imaging (MRI) angiography of the left lower limb was performed using spiral axial sections with coronal and sagittal reformatted images. MRI revealed diffuse T1 hypointense lesions extending across the fascial planes of the left thigh, knee, and leg involving both medial and lateral compartments. The entire left limb appeared hypertrophied, and multiple lobulated lesions were noted in the calf region, the largest measuring approximately 3.8x2.9cm. Scattered T1 hypointense foci, likely phleboliths, were observed within the lesions. The underlying bones, including the femur, tibia, fibula and ankle joints, demonstrated normal signal intensity with no evidence of marrow involvement or abnormal vascular flow voids. Vascular malformations are congenital anomalies of vascular development, classified based on their flow characteristics into slow-flow (venous, lymphatic or capillary) and high-flow (arteriovenous) lesions. Venous malformations are the most common type of slow-flow lesions, typically presenting as soft, compressible and non-pulsatile masses that may cause limb hypertrophy and contain phleboliths. MRI plays a crucial role in assessing the lesion´s extent, tissue involvement and hemodynamic pattern. In this case, imaging findings of diffuse T1 hypointense lesions with phleboliths and soft tissue hypertrophy were consistent with a slow-flow venous malformation involving the thigh and leg. Early recognition using MRI is crucial for accurate classification, multidisciplinary management planning and prevention of long-term complications such as chronic pain, bleeding or limb deformity.

**Figure 1:**
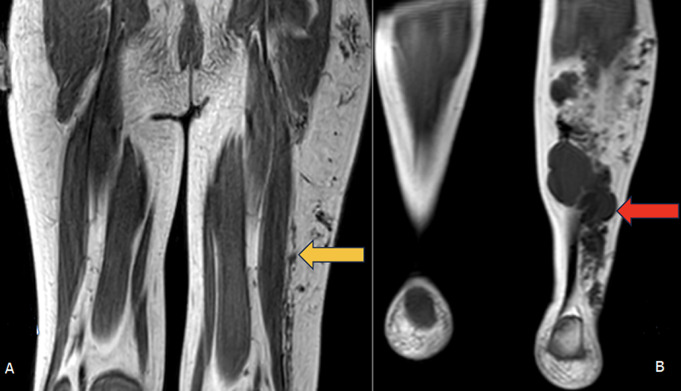
A) coronal T1-weighted MRI demonstrating diffuse hypertrophy of the left lower limb with hypointense vascular channels and phleboliths (yellow arrow); B) coronal T1-weighted MRI of the left lower limb showing multiple well-defined T1 hypointense lesions (red arrow) extending through fascial planes, consistent with venous malformation

